# Chemistry and Therapeutics

**Published:** 1884-11

**Authors:** John H. Logan

**Affiliations:** Professor of Chemistry, Atlanta, Ga.


### THE ATLANTA
### MEDICAL AND SURGICAL JOURNAL.

VOL. I. NOVEMBER, 1884. No. 9.

### Original Communications.

### CHEMISTRY AND THERAPEUTICS.

AN ADDRESS DELIVERED AT THE OPENING OF THE ATLANTA MEDICAL COLLEGE, OCTOBER 9, 1884.

BY JOHN H. LOGAN, M. D., PROFESSOR OF CHEMISTRY, ATLANTA, GA.

In the brief time allowed me to prepare for this occasion, I could find no subject, gentlemen, more interesting or profitable to you than the one I have chosen, viz., Chemistry and Therapeutics. It would be more correctly written, Sanitary Therapeutics, though at the risk of presenting a new coinage of word combination. But you must remember that the word therapeutics is generic, and includes under it several species: It treats not only of the administration of drugs, but of dietetics and vestment, and takes cognizance of hygiene, that department of medicine which respects the preservation of health. In all practical medicine there is not a broader nor more comprehensive branch than therapeutics, and I hope before reaching the conclusion of this address to show you that there is not one which equals in importance that department of it which I designate as sanitary therapeutics. But the essential basis of all therapeutics is chemistry, and hence the propriety of my heading—"Chemistry and Therapeutics."

It will be my principal aim to draw a broad distinction between the mere drugging therapeutist, the old Greek therapeutikos, the curer and the enlightened sanitary therapeutist, now but just emerging in our time from the prejudices, ignorance and false theories of the past. But in all the past there is one bright spot, one oasis in the surrounding desert on which I delight to linger in imagination while I gather courage and hope for the future. I refer to the age and practice of Hippocrates. No country, either ancient or modern, ever produced a greater physician than this illustrious descendant of Esculapius. Mankind do not erect temples and statues to the memory of men who lived only for themselves, to whose genius and benevolence they owe nothing; and no man could project his influence through more than 2,300 years, and thus impress himself upon remote future ages and not be deservedly illustrious. Has not the time come for living physicians to inquire earnestly into the causes of the extraordinary influence and success of this wonderful man? They appear to have been two-fold: first, his high moral character, his lofty integrity, and unselfish benevolence; second, the remarkable simplicity and good sense which regulated all his theories and guided him in his practice.

It is to the latter that I desire especially to direct your attention. One of his leading aphorisms was that little medicine should be given, particularly in acute diseases, his chief dependence being upon the powers of Nature in the restoration of lost health and functional energy, for it is to Hippocrates that modern medicine is indebted for the principle preserved in the old Latin formula, *vis medicatrix naturæ.* He moreover wrote learnedly on diet in acute diseases, anticipating by more than 2,000 years the discernment and discoveries of which our own boasted age is just now in the act of a tedious parturition, and bequeathed to mankind an invaluable treatise on Air, Water and Situation, a work covering almost the entire field of modern sanitation. We are told finally that he became so skilled in pathology and therapeutics that he has been regarded as the founder of medical science. It is important to add that he achieved all this success and reputation without a particle of anatomy or physiology, and with so meagre a knowledge of chemistry and materia medica as not to deserve the name of science. Let me be distinctly understood here. I do not mean by this last remark to yield an inch of ground to indolence or pretentious ignorance; it is not pertinent to my design, as will be seen, and besides, such minds as Newton and Hippocrates do not appear on earth except in vast millennial intervals. They were able to grasp truth by intuition; the great herd of mankind are obliged to exhaust every resource of knowledge and delve for dear life in order to be lifted to any respectable level of professional life.

With these facts, is it a matter for wonder that Hippocrates was so renowned? He stood at the very antipode of everything like quackery, was ridden by no hobby, and possessed a judgment far too profound to allow him to fall for a moment into visionary and impracticable schemes. Especially observe that he was remarkable for his knowledge and skill in sanitary, not drug therapeutics, which he had chiefly attained by making pathology a means to that important end. Moreover, observe that his system of therapeutics included not only air, water and situation, the environment, but dietetics and vesture as well, embracing the entire sanitary condition of patients and communities.

Happy, yea, thrice happy would it have been for the world had this Hippocratean philosophy been adhered to and developed through the succeeding centuries. Its direct tendency was to emancipate the race from the thraldom of disease, and to elevate the medical profession to unassailable heights of dignity and influence. Hippocrates was delving in the right direction through the whole of his long career, but alas! he was mortal.

Let me illustrate this idea of tendency and direction by a historical event which occurred during the war for independence in the immediate neighborhood of my boyhood home. The celebrated Polish patriot and exile, Kosciusko, was assailing as chief engineer under Gen. Greene a fort held by a British force. They had failed in a disastrous attempt to carry it by storm. It was then determined to approach it by sap, as Gen. Grant did our entrenchments at Petersburg, Virginia. A tunnel was run some thirty yards in length towards the centre of the redoubt, when the discovery was made that they were excavating in the wrong direction to reach the centre of the fortification. Kosciusko, not discouraged, went back a few yards, corrected his bearing, and had tunneled more than fifty yards in all, more than sufficient to have effected his purpose if he had not erred in his first direction, when the enemy, discovering his design, forced him by a counter excavation to abandon the work. Thus it is in medicine and in every art; it is essential to begin and contiue in the right course in order to achieve great results. Precisely analogous to Kosciusko's first tunnel at old Ninety-six has been the course of the profession in its long conflict with human ailments; it has persistently run its excavations in wrong directions in the vain attempt to reach the strongholds of the enemy. But since no comparisons must be pushed too far, it by no means follows that it too will be driven from the field in an attempt even now to change its base. Our instructors and practitioners have depended too much and too exclusively upon the mere teachings of anatomy, physiology, pathology and practice, to the almost total exclusion of chemistry and therapeutics, sanitary therapeutics. Of drugging, the profession cannot be justly charged with negligence. During all the dismally dark centuries intervening between the age of Hippocrates and the present, the drugging system has prevailed, and a faithful portraiture of the horrible substances and compounds and cruel processes often resorted to for the cure of disease, under the insane notion that every malady has its specific, would be too monstrous for belief, and too disgusting for a cultured audience to hear. Even the great Dr. Rush, philosophic as he was, believed and taught that a specific would yet be discovered for tuberculosis. A rational system of therapeutics, firmly based upon chemistry, effectually preventing, not curing disease, is the great desideratum of the profession and of the world. It is the only power that has ever won a real triumph for medicine, and yet, strange to say, it is still unrecognized as such by the multitude of drug compounders and specific venders of the age.

Sanitary therapeutics is immeasurably in the rear, practically, of all the other great branches of a complete medical curriculum, yet in fact it is the grand end of them all. It is the master builder, to which they are but the hod-carriers. They are the scaffolding, as it were, by which its beautiful temple is to be reared and beautified forever. What boots it, I ask, if we know every fiber, molecule and atom of the human body, all the laws of life, every shade of departure in color, consistency and function from the normal standard of health, and can apply every known agent in a routine practice at the bedside, and come far short, lamentably short of any reliable therapeutical results. To restore the patient to health and safety is the prime end of all medical knowledge, but this is just what the profession has failed in any satisfactory manner or system to do.

Do any of my brethren wince at this? Let them possess their souls in patience; like a good surgeon, I am cutting to do good. Even at this day, this enlightened day, blazing in the effulgence of all sorts of new lights, an age more than two thousand years in advance of that of the illustrious Greek of whom I have said so much, there are few events more uncertain than the recovery of a patient seriously ill and in the hands of the ablest practitioner among us. There is not a physician in this room who will not confess, if honest with himself, that when a patient is once assailed by a dangerous malady, he has set out on a run between Scylla and Charybdis— the doctor's drugs and management on the one hand, and the disease on the other. And if he should happily escape both and live, it would be impossible to say which had most to do with the result, nature or the doctor. This truth is embalmed in three remarkable proverbs, current respectively in the then leading nationalities of the world. "The doctor is more to be feared than the disease," says the French proverb. "It is God that cures and the doctor gets the money," runs the Spanish proverb. "The best physicians," says the old English proverb, "are Dr. Diet, Dr. Quiet, and Dr. Merry-man."

I am constrained to present now a far more tangible and damaging proof of the fact than these popular proverbs, and that is the universal and exceedingly prosperous existence of what we are pleased to call quackery. Did any one of you ever devote a half hour to the philosophical consideration of quackery? The subject, I assure you, gentlemen, is far more worthy of your careful study than a thousand plantations of Koch's micrococci. Newton sitting in his garden saw an apple fall to the ground. He instantly asked himself the question, Why did the apple fall? When you saw a quack rushing along the street, sack in had, did you ask yourself the question why that quack is here? If you did not, I will ask it for you: it is because the regular profession has utterly failed to satisfy the expectations and confidence of the people. Michelet, a recent French writer, remarks, in a popular book on woman, that if it were not for destitution there would not be a prostitute in France. Precisely analogous is the position of the irregular practitioner. If the profession could master the situation there would not be a quack in Georgia. Since being able to link him thus with a rational piylosophy, my respect for the quack and the vender of quack medicines has sensibly appreciated. One thing is certain, it ill becomes a physician to cast stones at the quack; every stone that strikes him, wounds the people at home.

These are evolution times. I once laughed and poked fun at the monkeys. I shall do so no more; it turns out that they are the old folks of the family. This makes it plain, too, why ignorance cannot be regarded as the mother of quackery, and why the cab of the irregular practitioner is seen standing as often before the palace as the cottage.

There are some remarkable points of resemblance as well as of difference between the two great rival classes of the healing art, which it will not be irrelevant or unprofitable for us to consider a moment. The regular profession physics the people. Quackery does more; it drugs and educates them as well. And this popular education through quackery is as alarming as it is universal. Ought the people to be censured for their well-known erroneous notions of medicine and its practice, when they are indoctrinated and their opinions formed by the teachings of quackery? Ask the average man, yea, and many far above the average, on these streets and in the country what his idea of disease is. He will tell you that it is some sort of hideous hydra-headed thing which gets into the bodies of men from without, and which it is the business of the doctor to kill or destroy by his magic drugs and nostrums. If he fails to do this after a fair trial, he is not a trustworthy doctor. Ask that white man, or the brother in black shoveling dirt yonder, what he thinks of calomel or other mineral drugs. He will tell you that they are poisons, to be discarded by all who would preserve life and health. Whence came these notions and prejudices? They were derived first from what may be aptly styled the way-side literature of quackery, from the advertisements, emblems, signs and pictorial devices emblazoning every accessible fence, plank, wall, tree, rock and house, in valleys, on mountains and plains, along every thoroughfare and byway reaching from Maine to California. They were imbibed from the illustrated pages of every newspaper, journal or magazine, whether scientific, religious or secular, published on the American continent. This voluminous flying literature speaks in a thousand cabalistic forms of hissing snakes, mysterious letters and monograms, and as a system of popular education is more potent and far-reaching in its influence upon the masses than all the educated physicians, medical colleges, academies and associations in every community, village, city or town in the United States.

With these facts, can we wonder, I repeat, that the people are so ignorant of the nature of disease, of the principles of medicine and of the character and obligations of the true physician? How can a well informed physician of this city, having his eyes open, believe that ignorance is the mother of quackery? Is not the contrary of the proposition the truth? Quackery is the mother of ignorance. More than half of the medical practice of this city is done by irregular doctors and patent medicine venders. There are more sectarian practitioners in Massachusetts to the thousand inhabitants than in Alabama. One of the most intelligent medical gentlemen I ever met, a native of Massachusetts and a graduate of a Northern university, remarked to me in my office that the admirable medical laws now in force in Alabama, could never be enacted in Massachusetts, because of the general dominancy of quackery in that State.

We are admonished, gentlemen, by all this to be patient, prudent, and modest. The sectarian doctor is our brother, after all, and there is no disputing our solidarity with him in many respects. He is after money and gets it. So are we, but we don't always get it. We drug our patients; so does he. He sometimes loses his; so do we. And when we meet him on the street or in the social circle, let us not play the Pharisee, and say to him, "Stand aside, for I am holier than thou;" but with the charity of the old Roman augurs, let us look him in the face, while he does the same with us, and mutually smile at the suggestions of unbidden thoughts. The law of the commercial world is, that demand and supply are inseparable and commensurate. When there is no more demand for the sectarian doctor, no more sectarian doctors will be forthcoming. But remember the power of reflex action, whether normal or abnormal—that quackery educates—is self-producing. The most hopeful sign now seen in the medical firmament is the incipient renaissance of the Hippocratean principles of sanitary therapeutics. This is tunneling in the right direction. It is the hope of the world's emancipation from the horrors and thraldom of epidemic disease, and an earnest of the profession's advancement to the dignity of a science and the perfection of a divine art.

From this source have come all the real triumphs of which medicine can boast, but yielding little credit to medical men, with the single exception of vaccination, discovered by Jenner in 1778. Permit me here to nail my theses to the wall, and then I shall proceed to defend them further by as many arguments and illustrations as I have time to present. I boldly declare,

1st. That the present status of medicine—I say nothing of surgery, its glory speaks for itself—is unsatisfactory, discreditable to the advanced age in which we live, and far below the expectations and hopes of the people; and that this inadequacy is due partly to inherent difficulties, and partly to the selfishness and narrow, false views of medical teachers themselves.

2d. That the only hope of improvement and success for the future is in an immediate return to rational Hippocratean therapeutics—the supreme advancement of chemistry and hygiene.

The first proposition requires no further proof than that already given. The first two examples that I shall adduce in support of the second are supplied by surgery, because they admirably suit my purpose. Velpeau, of France, was the greatest diagnostician of his time, it is said, in the world. When at the fullness of his reputation and practice in Paris, there came to the city a young man from one of the provinces to consult some surgeon in relation to a singular tumor which had always affected his scrotum. It was not inflamed nor the least painful; gave him no trouble in that regard. But he was about to marry, and ever since his engagement, it had been to him a source of anxiety that any malformation should exist in those parts. He passed at Paris through the hands of several of the most distinguished surgeons then in the metropolis, but they could make nothing of the case. It was a time of great professional rivalry between some of them and Velpeau. The young man at last came to Velpeau and was examined by him in the presence of a friend of mine, a distinguished Georgian, then a medical student in Paris, and from whose lips I received the story as I now write it.

Velpeau gave the patient a most careful investigation, but he too, was puzzled for a time. At length, rising to his feet, he exclaimed in his usual abrupt manner, "Gentlemen, this is a congenital, encysted fœtus or the devil himself." Frenchman-like, having made a diagnosis, the next step was to verify it. There was no other neeessity for an operation, and he was aware that gangrene at that time raged in the city hospitals. The very next day Velpeau insisted upon an operation, and actually prevailed upon the young man to yield to his wishes. The operation was a complete verification of Velpeau's remarkable diagnosis, and added another triumph to his professional glory. But the unfortunate patient, a splendid specimen of manly strength, fell a victim, after a few days, to hospital gangrene. There are multitudes of similar cases; pathology and diagnosis are made to take precedence of humanity and sanitary therapeutics, and for no other end than to gratify personal vanity and ambition.

My next illustration of the same proposition is Listerism. What a flutter and noise this simple introduction of a little wholesome sanitation in the management of wounds produced. True, Lister is delving practically in the right direction, but the objection to the system is the ridiculous parade it presents of a false theory in regard to the assumed causative existence of bacteria. In Germany particularly where, time out of mind, both people and surgeons had been assiduous, though unconscious, cultivators of the dreadful micrococci before their famous Koch was born, so great was its efficacy that it was hailed with joy. The detergent powers of the magic spray were seen to work wonders, and the nation was happy. This was well, but what a triumph for soap and water—for chemistry and sanitary therapeutics; for in no great while it was discovered that the same precautions of cleanliness without the germicides were followed by the same good results. Many distinguished operators in different countries who had heartily adopted the spray and carbolization of Lister, laid them aside, and are now content to undertake the most formidable operations with no other safeguard than strict cleanliness of the wound, instruments and surroundings. Listerism, after all, is nothing more than a late application of the principles of sanitary therapeutics. No doubt there are microbes hovering around every surgical operation either as cause or effect; who knows? But the chief concern of the surgeon is to obviate by his methods the conditions which have been recognized since the time of Hippocrates as productive of filth, vermin and disease. This whole micrococci mania reminds one of a Confederate soldier sitting leisurely down to catch, count, assort and bottle his camp lice, instead of using a few well known remedies to destroy them.

My next illustration is the immortal Koch himself. No other man has ever become so famous as he on so small a stock in hand. His labors have been cheered and welcomed in every enlightened country. Many of us present to-day entered with enthusiasm into the spirit of his novel investigations, fondly hoping that our Joshua had at last come to lead us out of the deserts. But alas, for the vanity of human hopes! We may well this morning exclaim, *cui bono!* For what good has come of it? Where is the practical outcome of Kochism? He has planted, propagated and studied as they were never studied before, whole generations of the bacilli of tuberculosis, and pursued the microbes of cholera with remorseless scrutiny into the strongholds of their own ancient habitat, yet people are dying to-day of phthisis and cholera just as they were wont to do before Koch and Pasteur were born.

The trust of the world was, that having discovered the germ origin of these terrible scourges of the race, there would remain but one more glorious step to find a germicide, which must destroy them forever. Has this expectation been met? Numerous investigators besides Koch and Pasteur—ardent, confident and equally able—went to work, particularly in France, to find a germicide for the bacillus. They cultivated generations of it, just as Koch did, and applied every known agent of disinfection. Their failure was as complete as it was discouraging. One or two drugs, such as mercuric-chloride, was discovered capable of destroying the bacillus, but not one with safety from toxic effects to the tuberculosed patient.

Such is the end of the promises of Koch. He excavated, I grant you, straight towards the citadel of the enemy; he was in the line of sanitary therapeutics, but his misfortune is that his ammunition was worthless when everything had been got ready for the explosion. It refused to go off at the touch of the match. But there is nothing without its uses. There is a profit in Kochism. It failed to find a specific for tuberculosis, but it has supplied another demonstration of the value of sanitary therapeutics. It teaches the transcendent importance of hygiene. A better hygiene is the outcome of Kochism.

My next illustration is from the history of yellow fever. The notion is quite prevalent that this epidemic scourge is indigenous in and peculiar to Southern latitudes. Facts do not sustain it. The first invasion of the United States by yellow fever was not in the South, but in Boston, at the very hub of the geographical wheel. This was in 1691, and two other invasions followed between that date and 1695; it reached Philadelphia, and between that year and 1794 this city suffered from eight other epidemic invasions of the disease. Never since has it once appeared as an epidemic in Philadelphia. The same pestilence reached New York in its southward march in 1702, and up to 1798 six other invasions had ravaged that city. New Haven was twice scourged by it between the years 1743 and 1794. It reached Providence, R. I., in the last-named year. From Providence it swept to Charleston, S. C., through Norfolk, Va., and raged in the former city through ten successive invasions before reaching New Orleans in 1796. Dr. Dowell, in his monograph on yellow fever, says it is an interesting fact in connection with the portability of the disease that New Orleans, though closely connected by its geographical position where yellow fever had prevailed for centuries under Spanish, English and French rule, was yet always exempt up to 1796.

Now, the point I wish to make in these interesting facts of yellow fever history is, that it has gradually and uniformly declined, and at length ceased to exist except sporadically, here and there in all places of the United States where it once raged epidemically, northward, at least of Charleston, S. C., and moreover that this declension is now in progress in all the cities of the South, through the judicious application of the principles of sanitary therapeutics. The disease has almost ceased to invade even New Orleans, and many of its most enlightened health officers regard its further prevalence in that city as an epidemic impossible.

Is there not in all this a lesson crying in thunder-tones to people who are supposed to be endowed with common sense? What did the great National Board of Health effect by all their assinine talk and bluster about the yellow fever microbe? What do the sensible people of Boston and Philadelphia care for the yellow fever micrococci, when they have been rid of the invasions of the pest more than a century before they knew that it had any cocci?

In contrast with these happy results, effected by the quiet use of sanitary therapeutics, permit me to present a sample of the drug conflict with this fell disease in the various stages of its march. In the first place, no two of the doctors agree as to the real pathology of the disease. What must be the jarring, then, when they meet in consultation about its treatment! One, regarding it a malarious disease purely, pours in the mercury even to ptyalism. Another, perceiving, as he thinks, periodicity in its exacerbations, cries out for quinine, and shovels in the alkaloid by the drachm. Still another, observing its blood changes, its spanemia, its hemorrhagic character, cries as lustily for iron; iron is the great hæmostatic remedy. A fourth wiseacre sees no indications, according to his theory, for any of the above-named drugs, but for turpentine, and forthwith turpentine is administered to the bitter end, both inside and outside. A fifth puts his whole reliance in acids, the free use of lemon and lime juice. And lastly, a sixth professional Solomon proclaims the stomach to be already too full of acidity, and dumps in the alkalies *usque ad nauseam.*

The result of this confessed ignorance is the prompt death of every patient whose strength of constitution is unequal to the conflict with the drugs and the disease. There is a grim consolation in the following words from a Texas doctor, D. T. R. Wallace, of Waco: "Possess your spirits in patience, friends. It is no worse in this than in other diseases. It only seems so because a new disease, draped in obscurity. You are over anxious for certitude. Discard the expectation—it belongs not to the medical art. Specifics are but the dreams of distempered imaginations. Our bodies are too wonderfully made for any well-informed medical man, in the present condition of our science, to think twice of the possibility of specifics. The empiric, and he only naming his disease, plies his specifics."

My last argument is taken from the history of the Plague, the dreadful epidemic which for so many centuries desolated all Europe. The terrors of this disease were appalling. From its first appearance, about the time of Mahomet's rise in the sixth century, A. D., it ravaged Europe and the East for more than twelve hundred years, destroying vast multitudes of the race, and often reducing entire cities and provinces to hopeless ruin. No doubt this fell pest had its microbes like tuberculosis and cholera, but there was no Koch there to cultivate and make them famous. There were myriads of Agamemnons, but alas! no Homer. And I venture the assertion that had such an investigation as a Koch would have given them in their habitat been the only reliance of the miserable people for deliverance, epidemic plague would be a scourge of Europe to-day. A merciful God had something better in store for his suffering children. It was the habit of the pestilence to resume its desolating marches after intervals of thirty or forty years. Its last visit to London and England occurred in 1665, and the event by which it was crushed out forever, at this date in the even then overgrown English metropolis, is one of the most remarkable in the annals of any people. Towards the close of that year the ravages of the epidemic had been unprecedented in severity. The king, with his court and all who were able, fled the death-invaded city, leaving the poor and unfortunate to the mercy of the destroyer. 1666 dawned upon London a desolation and ruin. It was at this auspicious juncture that God in his wisdom as well as mercy sent an angel and burned the deserted city to ashes, scarcely a house was left. And shortly afterwards he sent another angel, who inspired the city fathers and board of health with a sufficiency of common sense to rebuild new London with broader streets and better ventilated houses.

The happy results were worth the stupendous sacrifice. From that day to the present, more than two hundred years, there has not been another case of plague in London, and by 1816 it had disappeared forever from the whole of Europe. That conflagration—the sublimest display of chemical action, and the grandest application of sanitary therapeutics the world had ever witnessed—made a clean sweep of the micro-organisms of plague. They will never again breed in the same habitat, unless some English Koch, more cunning than his German colaborer, shall recall to life out of their carbonized dust enough of the race to form a new London colony.

If time permitted I would add another argument no less interesting and cogent for my purpose from the history of vaccination and variola. It is sufficient only to name the glorious triumph of Wm. Jenner in sanitary therapeutics. And now since 1798 how vast the obligations of the profession and the world to him, who, by his genius and energy, introduced the incomparably better and safer method of inoculation with the mild vaccinia, instead of the old one of drugging, in dismal uncertainty, the more deadly variola. The change was like a new creation. And yet, though the micro organisms of vaccine lymph were present in myriads, no microscopic revelations were attempted or thought of; not the shadow of a micrococcus disturbed the dreams of the immortal Jenner. Well was it for the world and the cause of humanity that it did not.

Gentlemen, I am done, after too long a trial of your patience. And in the conclusion, as at the beginning, I assert my profound belief that the true dependence of the profession in its successful march to scientific exactitude, and to the fulfillment of the people's hopes in their unutterable heart longings for deliverance from the thraldom of disease, are not chiefly anatomy, nor physiology, nor pathology, nor practice, nor drugs, nor specifics, nor all these combined, but chemistry and sanitary therapeutics.

